# Enhancing Heart Failure Diagnosis Accuracy and Distinguishing It From Other Pulmonary Conditions: A Retrospective Case Series Study Leveraging the HeartLogic Parameters

**DOI:** 10.7759/cureus.65949

**Published:** 2024-08-01

**Authors:** Kamran Zaheer, Bruno Goncalves, Mohamed Suliman, Archana Ramalingam, Komal Sodhi, Carlos Rueda Rios

**Affiliations:** 1 Internal Medicine, Marshall University Joan C. Edwards School of Medicine, Huntington, USA; 2 Surgery and Biomedical Sciences, Marshall University Joan C. Edwards School of Medicine, Huntington, USA; 3 Cardiovascular Medicine, Marshall University Joan C. Edwards School of Medicine, Huntington, USA; 4 Surgery and Biomedical Sciences, Marshall University, Joan C. Edwards School of Medicine, Huntington, USA

**Keywords:** rural setting, heartlogic, pulmonary diseases, heart failure, heartlogic index

## Abstract

Introduction

Heart failure (HF) poses a substantial and escalating medical and economic challenge, marked by significant morbidity and mortality. It stands as the primary cause of hospital admissions among the elderly, contributing significantly to healthcare expenditures in developed nations. Evaluating cardiac and pulmonary function remains challenging, necessitating careful interpretation to mitigate misdiagnosis and inappropriate treatment. Remote monitoring has emerged as a preventive strategy to curb HF-related hospitalizations, emphasizing the importance of early detection of impending acute HF decompensation. Implantable cardiac defibrillators (ICDs) capture various parameters, including heart rhythm, pacing percentages, thoracic impedance, and physical activity.

Objective

In this study, we aim to investigate the effectiveness of HeartLogic (Boston Scientific, Marlborough, Massachusetts) parameters in accurately distinguishing HF patients from individuals with alternative diagnoses.

Methods

This cross-sectional study was conducted at Cabell Huntington Hospital, St. Mary's Medical Center in Huntington, West Virginia, between 2021 and 2022. The study involved a retrospective chart review of electronic medical records, approved by the institutional review board, encompassing patients aged >18 admitted with Heartlogic-capable devices. The analysis included demographic variables, admission and discharge diagnoses, length of hospital stays, health literacy index, and thoracic impedance.

Results

Of the initially included 26 patients, 19 met all inclusion criteria. The demographic profile highlighted a predominantly older population with a male preponderance and a notable incidence of congestive heart failure (CHF). Physiological changes, particularly in thoracic impedance and the HeartLogic Index, demonstrated significant alterations. Logistic regression analysis revealed that changes in health literacy index and thoracic impedance significantly contributed to predicting the change in CHF diagnosis.

Conclusion

This study, conducted in a rural setting, demonstrates the capability of the HeartLogic algorithm in predicting HF events, providing valuable insights into its utility in diverse clinical environments. The findings emphasize the potential of this technology to enhance diagnostic accuracy and improve patient outcomes. Despite inherent limitations, this analysis contributes unique perspectives, particularly in the context of a specific and underexplored rural population in West Virginia.

## Introduction

Heart failure (HF) represents a prominent and escalating medical and economic challenge, marked by substantial morbidity and mortality [1, 2]. HF is the first cause of hospital admission among the older population and is the leading cause of death, accounting for 1-3% of the total healthcare financial burden in developed countries [2, 3]. Projected HF-related costs in the United States are anticipated to soar to 70 billion dollars by 2023, reflecting a 130% surge from current expenditures [4].

Assessing cardiac and pulmonary function often presents challenges fraught with occasional misleading outcomes. Careful interpretation is required to avoid misdiagnosis and inappropriate treatment [5]. In the past decade, a mounting interest has been in remote monitoring to forestall HF-related hospitalizations [6]. The timely detection of impending acute HF decompensation holds paramount significance, offering the potential for outpatient interventions before severe decompensation occurs, thereby mitigating HF hospitalizations and associated costs [7, 8]. Parameters that are sensed by and stored on the implantable cardiac defibrillators (ICDs) encompass heart rhythm, pacing percentages, thoracic impedance, and physical activity, among others [9].

In response to the diagnostic intricacies inherent in HF, advanced monitoring systems like HeartLogic (Boston Scientific, Marlborough, Massachusetts) have emerged as promising tools to enhance the diagnosis and management of this condition. These systems leverage innovative parameters and algorithms, providing comprehensive and continuous cardiac function monitoring [7, 9]. It combines heart sounds (S1 and S3), respiration rate, thoracic impedance, nightly heart rate, and physical activity [8]. However, it is critical to validate these parameters' efficacy and accuracy in differentiating HF from other conditions, especially in diverse clinical settings.

Rural populations encounter distinct issues regarding the availability and accessibility of high-quality healthcare services. Compared to urban residents, rural individuals exhibit a higher incidence of cardiovascular disease and a greater likelihood of experiencing complications related to congestive heart failure (CHF), leading to increased readmissions [10]. It has been shown that rural inhabitants are less likely to receive regular preventive care, resulting in fewer medical visits, underdiagnosis, and suboptimal health outcomes [10, 11]. Studying our series of cases in a rural setting, we sought to share our unique experience and highlight the importance of evaluating the performance of advanced monitoring systems like Heartlogic in diverse clinical settings. This study, therefore, aims to investigate the effectiveness of Heartlogic parameters in accurately distinguishing HF patients from individuals with alternative diagnoses. The data from this study could provide relevant perceptions into the utility and potential of HeartLogic for improving HF diagnosis and patient outcomes.

## Materials and methods

Study design

This cross-sectional study was performed at Cabell Huntington Hospital, St. Mary's Medical Center in Huntington, West Virginia (WV) between 2021 and 2022. The study methodology entailed a meticulous retrospective examination of patients' electronic medical records (EMR) who had received the HeartLogic device (Boston Scientific, Marlborough, Massachusetts) placed on the chest. Approval for the study protocol was obtained from the Institutional Review Board (IRB) of Marshall University Joan C. Edwards School of Medicine (IRB NO: 2001324-1), and a waiver for informed consent was granted.

Patient selection and data collection

Trained hospital personnel examined patients' electronic medical records (EMRs) with strict confidentiality measures per the Health Insurance Portability and Accountability Act of 1996 (HIPAA). Patients were identified by reviewing EMRs from 2021 to 2022; eligible participants were those over 18 years old and admitted to the hospital with a HeartLogic-capable device. Exclusion criteria included patients under 18 years old, pregnant individuals, and those with missing or incomplete Heartlogic data. Data collected from eligible patients included variables such as age, gender, admission diagnosis of CHF, discharge diagnosis of CHF, length of stay in the hospital (LOS), health literacy (HL) index, and thoracic impedance (TI).

HeartLogic Index

HeartLogic is a proprietary algorithm that utilizes data from various implanted sensors within a commercially available cardiac resynchronization therapy defibrillator, designed to target multiple facets of HF pathophysiology associated with common signs and symptoms. The algorithm extracts trends from sensors, including the first and third heart sounds, respiration rate, rapid shallow breathing index, thoracic impedance, heart rate, and patient activity. Changes in sensor data from the patient's baseline are aggregated and weighted to determine an individual's daily risk, culminating in calculating the daily HeartLogic HF index.

Statistical analysis

Data were analyzed using SPSS version 26 (IBM Inc., Armonk, New York). Demographic and clinical variables are presented as frequency/percentage. Baseline characteristics between the Heartlogic and control group were compared with the Chi-squared test for discrete variables. Data comparisons are presented with significance levels of p<0.05.

## Results

Initially, 26 patients were included in the study. Due to missing or incomplete data, 19 patients met all inclusion criteria. These patients, selected from a specific rural population, provided relevant perceptions of this demographic. Table 1 summarizes the basic characteristics and comorbidities of the study population in each group. Participants were categorized into two age groups, with the majority (57.9%) being over 66 years old. The study had a higher representation of males (68.4%). Regarding admission diagnoses, 89.5% of participants had CHF. For discharge diagnoses, 42.1% had CHF, while others had chronic obstructive pulmonary disease (COPD) and pneumonia. A significant portion of the patients (78.9%) had a hospital stay of less than six days. This comprehensive demographic dataset is essential for understanding the characteristics of the study population and will be used to assess the effectiveness of HeartLogic parameters in accurately distinguishing HF patients from individuals with alternative diagnoses.

**Table 1:** Summary of patient demographics and general clinical profile CHF - congestive heart failure

Category	Frequency (N=19)	Percentage (%)
Age (>66)	11	57.9
Gender (Male)	13	68.4
Admission diagnosis of CHF	17	89.5
Discharge diagnosis of CHF	8	42.1
Length of stay in hospital (<6)	15	78.9

In terms of physiological changes (Table 2), no participants exhibited a change in thoracic impedance within the range of 0-20. However, 31.6% experienced a change in impedance between 21-40, and a significant majority (68.4%) had a change greater than 40. Additionally, changes in the Heartlogic Index were analyzed, revealing that 57.9% had a change between 0-20, 26.3% had a change between 21-40, and 15.8% had a change greater than 40.

**Table 2:** Change in HeartLogic Index and thoracic impudence from baseline

Category	Change in HeartLogic Index from baseline (%)	Change in thoracic impedance (%)
0-20	57.9	0
21-40	26.3	31.6
>40	15.8	68.4

Table 3 provides the results of likelihood ratio tests used to assess the model fitting criteria for predicting the change in the diagnosis of congestive heart failure (CHF). The logistic regression model incorporates independent variables, including changes in the HL index, changes in thoracic impedance (TI), length of stay (LOS) group, age group, and patient gender, while the dependent variable was changed in the diagnosis of CHF. Chi-squared statistics were computed by comparing the -2 log-likelihoods between the two models. The LOS group does not reach statistical significance (p=0.189). Our data showed the same results for age and patient gender (p=0.136 and p=0.085, respectively). The change in the HL Index significantly contributes to the model fit, as indicated by a highly significant Chi-squared value of 18.423 (p<0.001). The change in TI also significantly improves the model fit, with a chi-square value of 11.968 (p=0.003), differentiating heart failure from other diagnoses. An interesting finding in our case series was that COPD was one of the most common misdiagnoses on admission, often mistaken for CHF. Among the 17 cases initially diagnosed with CHF, five were later determined to have COPD. The results suggest that the inclusion of the HL Index and change in TI significantly enhances the model’s ability to predict the change in the diagnosis of CHF. The other variables, although not all statistically significant, contribute to the overall model fit, providing valuable insights into their potential influence on the outcome.

**Table 3:** Likelihood ratio tests for model fitting criteria in predicting change in diagnosis of congestive heart failure (CHF) HL - health literacy; TI - thoracic impedance; LOS - length of stay

Effect	Model fitting criteria	Likelihood ratio tests		
	-2 Log likelihood of reduced model	Chi-squared	df	Sig.
Intercept	3.244^a^	.000	0	.
HL index change	18.423	15.179	2	<0.001
Change in TI	11.968	8.724	1	0.003
LOS group	4.970	1.726	1	0.189
Age group	5.462	2.218	1	0.136
Patient gender	6.206	2.962	1	0.085

## Discussion

HF and COPD are global epidemics incurring significant morbidity and mortality [1, 2]. The combination presents many diagnostic challenges. Clinical symptoms and signs frequently overlap. Evaluation of cardiac and pulmonary function is often problematic and occasionally misleading. Implantable technologies have been developed to detect HF early and accurately predict HF events, which is crucial for timely intervention and optimal patient outcomes [5]. Previous studies have shown promise in using the HeartLogic algorithm for this purpose as it has combined five pathophysiological CIED-based sensors: the first and the third heart sounds (S1 and S3, respectively), and the ratio of S3/S1, respiration rate, intrathoracic impedance, night heart rate, and physical activity to detect impending fluid retention [9, 12]. Each of these parameters is critical in differentiating HF from pulmonary conditions.

It has been shown that the S3 heard sound is a hallmark of HF, indicating increased ventricular filling pressures, whereas respiratory rate and intrathoracic impedance are sensitive to both cardiac and pulmonary congestion [13, 14]. The algorithm's ability to integrate and weigh these diverse signals allows for a nuanced differentiation between HF and pulmonary pathologies [15, 16]. This multifactorial analysis is crucial, as it considers the overlapping symptomatology and the complex interplay between cardiac and pulmonary systems. By refining the interpretation of sensor data, the HeartLogic algorithm can more accurately predict HF events and distinguish them from pulmonary causes, thereby improving diagnostic precision and enabling timely therapeutic interventions.

Our data showed that the HeartLogic algorithm demonstrated its capability to predict HF events. The HL index, which represents the algorithm's predictive ability, showed varying levels of change from baseline, indicating sensitivity to individual patient characteristics. Changes in TI values further supported the algorithm's predictive accuracy, with significant alterations observed in some cases.

This scientific inquiry substantiates our finding within the context of the prevailing research on the clinical applicability of the HeartLogic algorithm in the management of HF. A multinational MUSIC study provided empirical evidence of the HeartLogic algorithm's precision in predicting HF. The investigations highlight the diagnostic accuracy of the HeartLogic algorithm, revealing a heightened sensitivity (70.6%) and specificity (99.3%) in identifying impending heart failure decompensation within a real-world clinical milieu. Furthermore, a study conducted a retrospective analysis, affirming that the utilization of HeartLogic parameters significantly diminishes heart failure-related hospitalizations [9]. Their work, including the MultiSENSE study, accentuates the algorithm's prowess in predicting HF events among patients with implanted devices.

While the efficacy of the HeartLogic system has been demonstrated in various settings, there remains a need to validate its performance in diverse clinical environments, including rural settings. Rural populations often face unique challenges in accessing healthcare resources and expertise, necessitating tailored diagnostic approaches [10, 11]. A recent study has conducted a propensity-matched cohort analysis and reported fewer worsening HF events with the implementation of HeartLogic in standard care [17]. In addition, it is demonstrated that the algorithm's ability to detect fluid retention early and provide opportunities for timely intervention aligns with the primary goal of HF management [16]. Furthermore, the clinical and economic impact of HeartLogic compared to standard care showed reductions in HF-related hospitalizations and healthcare costs [8].

However, the study has inherent biases and limitations due to its retrospective nature and reliance on medical records for data collection. Prospective studies with standardized data collection protocols would provide more accurate and reliable data. Additionally, our study focused on short-term outcomes, and long-term follow-up is necessary to evaluate patient outcomes such as mortality rates and quality of life measures. Despite these limitations, this study presents important strengths. To our knowledge, no previous study has investigated the effectiveness of HeartLogic parameters in accurately distinguishing HF patients from individuals with alternative diagnoses in West Virginia. Therefore, we believe that the unique nature of the rural population studied is extremely important, providing valuable information on a specific demographic group that is understudied.

## Conclusions

In conclusion, HF is a prevalent and burdensome condition requiring precise diagnosis for effective management and improved patient outcomes. Our unique experience adds to the existing body of evidence and underscores the importance of evaluating the performance of such systems in diverse clinical settings. Further investigations are warranted to validate these findings and inform clinical practice guidelines. However, the use of innovative technologies, such as the HeartLogic algorithm, will be essential to optimizing therapeutic strategies to increase survival rates in this context.
